# Pitting Is Not Only a Measure of Oedema Presence: Using High-Frequency Ultrasound to Guide Pitting Test Standardisation for Assessment of Lymphoedema

**DOI:** 10.3390/diagnostics14151645

**Published:** 2024-07-30

**Authors:** Jennifer Sanderson, Neil Tuttle, Robyn Box, Hildegard Reul-Hirche, E-Liisa Laakso

**Affiliations:** 1 Menzies Health Institute Queensland, Griffith University, Gold Coast, QLD 4215, Australia; liisa.laakso@mater.uq.edu.au; 2 School of Health Sciences and Social Work, Griffith University, Brisbane, QLD 4111, Australia; n.tuttle@griffith.edu.au (N.T.); hildegard.reul-hirche@griffithuni.edu.au (H.R.-H.); 3 QLD Lymphoedema and Breast Oncology Physiotherapy, Brisbane, QLD 4051, Australia; 4 Royal Brisbane and Women’s Hospital, Brisbane, QLD 4029, Australia; 5 Mater Research Institute, University of Queensland, South Brisbane, QLD 4101, Australia

**Keywords:** lymphoedema, pitting, pitting test, pitting oedema, ultrasound, assessment, tissue composition, test standardisation

## Abstract

The pitting qualities of lymphoedema tissue change with disease progression. However, little is known about the underlying tissue response to the pitting test or the tissue characteristics that enhance or resist indentation. The pitting test is currently unstandardised, and the influence of test technique on pitting outcomes is unknown. Understanding how tissue reacts to applied pressure will build evidence for the standardisation of the pitting test. Ninety pitting test sites from fifteen women with unilateral breast cancer-related lymphoedema were evaluated using high-frequency ultrasound (HFUS), bioelectrical impedance spectroscopy (BIS), and limb volume measures. Three sites on each lymphoedema and non-lymphoedema arm were subject to a 60-s (s) staged pitting test, with changes in tissue features captured with ultrasound imaging before, throughout, and after the pitting test. Pitting qualities of tissues varied greatly, with lymphoedema sites pitting more frequently (*p* < 0.001) with greater depth (*p* < 0.001) and requiring a longer recovery time (*p* = 0.002) than contralateral unaffected tissue. Pitting is not solely attributable to oedema volume. Non-structural and structural characteristics of dermal and subcutaneous layers also influence tissue responses to sustained pressure. To enhance the validity and reliability of pitting assessment, a 60 s staged pitting test with an observation of tissue recovery is recommended for lymphoedema presentations.

## 1. Introduction

Pitting is integrated into several diagnostic and staging criteria as a characteristic feature of tissues that present with oedema [1,2]. Despite being a diagnostic outcome, the assessment of pitting remains unstandardised in healthcare, with great variability in the methods recommended and performed [3]. In the current unstandardised form, the authors suggest that the validity of the pitting test as a diagnostic or assessment criterion is unclear [3].

The research aims to understand the involvement of dermal and subcutaneous tissue layers in pitting, identify contributing factors to pitting depth, and guide the standardisation of the pitting test for lymphoedema assessment. The hypothesis was that a standardised pitting test method would yield more information than oedema presence or absence.

Pitting assessment is typically performed with sustained pressure of the thumb or fingers, with pitting defined as a visible indentation occurring on the skin [3]. A positive pitting result is interpreted as the presence of oedema, and the depth of pitting is recognized as an indicator of the relative amount of oedema [4]. The pitting attributes of tissues have been suggested to relate to oedema within the subcutaneous tissue layer [5], although the contribution of dermal and subcutaneous tissue layer features that promote or resist pitting is yet to be explored in research. Limited knowledge exists regarding the characteristic features of pitting tissue and the influence of the pitting test technique on pitting outcomes.

Multiple conditions are known to exhibit pitting, although the manner of pitting observed between conditions can differ. In some conditions, pitting tissue indents as pressure is applied, with an evident indentation produced within a few seconds. Pitting of lymphoedematous tissue is recognised to change with disease progression, from evident pitting in early and mid-stage disease to a loss of pitting in late-stage disease [2]. The change in pitting is generally accepted to derive from pathophysiological changes to tissue composition with respect to free-fluid, fibrotic, and adipose deposits [6]. However, how specific components of the tissue layers responds to sustained pressure has not been verified in research. In lymphoedematous tissue, there can be a delay in response to sustained localised pressure, whereby tissue pitting indentation can take over 4 s to initiate [7], and the soft tissue requires a long duration to adjust to the applied pressure [8]. 

There is limited evidence regarding how to perform the pitting test in various conditions. Concerning the duration of sustained pressure, the literature ranges in recommendations from 5 to 60 s [9,10,11]. The duration of applied pressure is critical for obtaining a consistent pitting technique as the pitting outcomes observed after a 5 s test are expected to differ from a 60 s test. 

The impact of the pitting test being unstandardised is that there is a variation in method and potentially an inconsistent interpretation of the pitting outcome. A previous study found significant variations in how the pitting test was performed in terms of pressure applied, the contact area of the thumb used to apply pressure, and test duration [3]. Diversity in the test method is unlikely to yield repeatable and reliable inter- or intra-rater results [3]. This research utilises high-frequency ultrasound to investigate how lymphoedema tissue responds to the pitting test, to understand the influence of the pitting test technique on pitting test outcomes, and to build evidence for standardisation of the pitting test.

## 2. Materials and Methods

Fifteen women with unilateral breast cancer-related lymphoedema (BCRL) were recruited to participate in the study. The unilateral BCRL cohort is ideal for comparative ultrasound research as fewer confounding factors can affect arm lymphoedema compared to leg presentations, such as venous insufficiency, which also presents with oedema. Ninety pitting assessments were performed by the same assessor across all participants in this study. Exclusion criteria were pregnancy, pacemaker, multiple limb lymphoedema, an incomplete first line of cancer treatment, or within three months of undergoing chemotherapy. 

Human Research Ethics Committee approval was received from Griffith University, Gold Coast (#2016/353) and the Centre for Advanced Imaging, The University of Queensland, Brisbane (#2016000887). This publication draws on data from the same participants in a publication detailing the Localised Objective Characterisation Assessment of Lymphoedema (LOCAL) method used to characterise test sites [12]. 

High-frequency greyscale ultrasound imaging was used to evaluate the dermal and subcutaneous tissue layers before, throughout, and after the pitting test. 

### 2.1. Pitting Test Method

Pitting assessment was performed using a staged method, modelled on techniques used by experienced lymphoedema therapists, as observed in an earlier study [3]. 

Using a broad thumbprint of 5 cm^2^, a force of approximately 5 kg was applied, equating to a pressure range of 11–14 Newtons/cm^2^. The pressure range is within the upper limits of our previously published work [3] and corresponds with findings from preliminary testing, which indicated firmer pressure is more reliable for the assessor to target than lighter pressure. Note that firm pressure is better tolerated by the participant when applied to dense tissue compared to soft tissue, and adjustments in the pitting technique occurred to ensure the pressure remained firm but did not cause pain to the participant. 

The total pressure duration was 60 s, comprised of three intermediate timepoints where the thumb was raised for a few seconds for ultrasound imaging. During the staged pitting test technique, the images were captured at the 10, 30, and 60 s timepoints. The technique of lifting the thumb throughout the pressure phase is consistent with what some experienced therapists use in clinical practice [3]. Where therapists evaluate tissue response using visual and palpation cues, this study utilised ultrasound imaging to measure change. Tissue layer recovery was observed for 4 min after pressure release with ultrasound images taken at 30 s intervals. 

Lymphoedema-affected and matched contralateral unaffected sites were assessed on each individual at predetermined distances proximal to the ulna styloid. The locations included the anterior forearm at 15 cm, the posterior forearm at 15 cm, and the posteromedial upper arm at 30 cm. Forearm sites were measured with the participant seated, and the upper arm site required the participant to be in a prone position.

### 2.2. Ultrasound Measurement

The Siemens Acuson S3000 (Siemens, Germany) ultrasound device with an 18 MHz linear transducer was utilised for high-quality greyscale imaging. All images were captured with a 2 cm standoff in place (2 cm × 9 cm Ultrasound Gel Pad, Aquaflex, Parker Laboratories, Fairfield, NJ, USA). Ultrasound gel was used as a medium between the three surfaces of the transducer, standoff, and skin. 

Greyscale ultrasound images were analysed using post-imaging processing software ImageJ version 1.50i (U.S. National Institutes of Health, Bethesda, MD, USA, https://imagej.nih.gov/ij/, 1997–2018, accessed on 15 July 2016). The test zone is the region where the assessor’s thumb applied the pitting test to the tissue. Within each ultrasound image of the tissue review series, measures were recorded from within the test zone and from outside the test zone to indicate local changes.

Measures included echogenicity and cross-sectional tissue layer thickness measurements. The thickness measurements were utilised to determine pitting depth, tissue layer involvement in pitting, and tissue recovery after pressure was released. Pitting was defined as an observable change in tissue layer thickness within the test zone. Recovery was defined as a return to homogenous ultrasound echogenicity with undisturbed tissue layer thickness.

Using pre-test images, the dermal-subcutaneous (d-s) border integrity was described subjectively for each test location by visual observation. The descriptions included a clearly defined border, slight border clarity deterioration, and evident deterioration of border integrity.

### 2.3. Bioelectrical Impedance Spectroscopy (BIS)

The Impedimed SFB7 device (ImpediMed, Brisbane, Australia) was used to obtain BIS measurements of R0 for upper and lower arm segments. Refer to the companion article for details on the method [12].

### 2.4. Lymphoedema Tissue Categorisation

The dataset is categorised using the Localised Objective Characterisation Assessment of Lymphoedema (LOCAL), which is a tissue classification presented in an article by the same authors [12]. The LOCAL utilises a contralateral comparative assessment site to define four categories of lymphoedema tissue characteristics: No Oedema, Early Fluid, Fibro-fatty change with High Fluid volume (High Fluid+), and Fibro-fatty change with Low Fluid volume (Low Fluid+). Objective measurement thresholds define each category for segment volume, bioelectrical impedance spectroscopy, and ultrasound image echogenicity of the dermal and subcutaneous tissue layers [12].

### 2.5. Statistical Analysis

Statistical analyses were performed using IBM SPSS version 25 (IBM Corp., Armonk, NY, USA). Descriptive statistics were used to report tissue layer characteristics; the Kruskal–Wallis Test was used to test the relationship between pitting qualities and LOCAL categories; Spearman’s correlation was used to test the association between pitting depth and indicators of oedema, including BIS segmental R0 ratio, percentage difference in segmental volume, and tissue layer thickness before the pitting test; and the Wilcoxon Signed Rank Test was used to compare lymphoedema-affected and unaffected results.

## 3. Results

Participants ranged in age from 39 to 70 years (mean 58.8 years). Participants had received surgical treatment including mastectomy (66.7%), breast-conserving surgery (33.3%), axillary dissection (86.7%), or sentinel node biopsy (13.3%). Eighty percent of participants received post-operative adjuvant cancer treatment in the form of either or both chemotherapy and radiation therapy. The time since surgery and lymphoedema onset ranged between <1 year and 10 years, with 73.3% of participants reporting the onset of lymphoedema occurring during the primary oncology treatment period.

Lymphoedema tissue demonstrated a pitting effect twice as frequently as matched unaffected sites, with significantly greater pitting depths when pitting occurred (*p* < 0.001) and a longer recovery duration than matched unaffected sites (*p* = 0.002).

**Figure 1:**
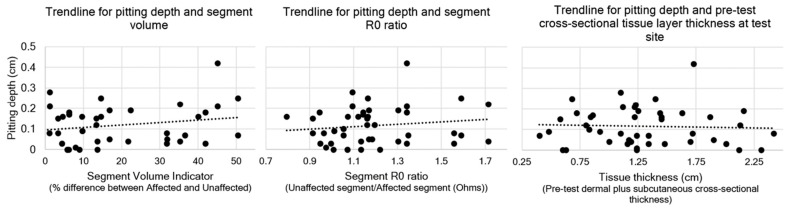
Trendlines for pitting depth and segment volume, segment R0 ratio, and tissue thickness. Dots indicate the individual results, trendline indicated by dotted line. Traditional indicators of oedema do not correlate with pitting depth. It is reasonable to infer that other factors contribute to tissue pitting.

With the ultrasound standoff in situ, the deepest total pit recorded was on the posterior forearm with a thickness change of 4.16 mm, comprising a dermal pit of 0.82 mm and a subcutis pit of 3.34 mm. The pitting depth of lymphoedema sites did not correlate with the segmental BIS R0 values (rs = 0.130, *p* = 0.395), cross-sectional tissue layer thickness analyses (rs = −0.72, *p* = 0.639), or segmental volume difference (%) (rs = 0.142, *p* = 0.352) (Figure 1).

The timepoint of initiation of pitting varied between sites throughout the pitting test pressure phase. Sites that registered a pitting response at the 10 or 30 s imaging timepoint were observed to increase pitting depth throughout the remainder of the test. In contrast, tissues that only displayed a pit at the 60 s timepoint had relatively shallow pitting depths.

**Figure 2:**
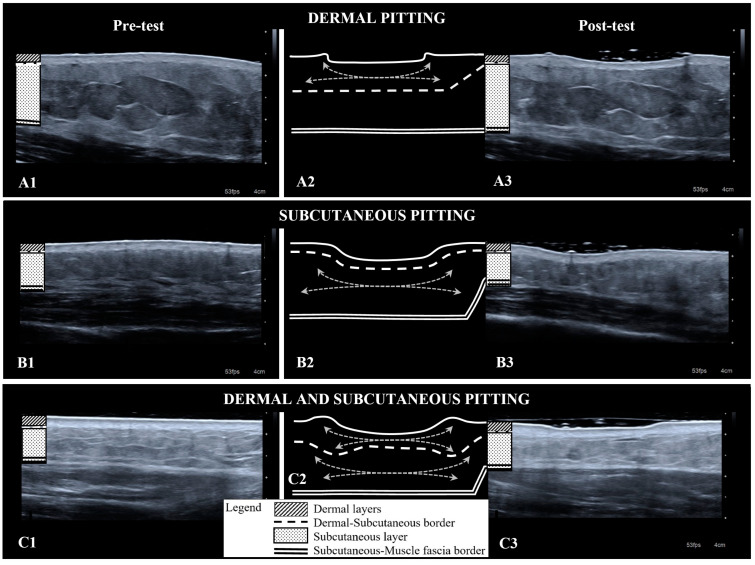
Ultrasound images and illustrations displaying tissue layer involvement in pitting. Pre-test ultrasound images on the left correspond with post-pitting test images on the right, taken with the weight of the standoff in situ. The diagrammatic representation of the tissue layers post-test is on a larger scale to illustrate tissue appearance without the weight of the standoff: skin surface (solid line), dermal-subcutaneous border (dotted line), muscle fascia border (double line) and fluid movement within the tissue layers (grey arrows). Dermal pitting: (**A1**) pre-test ultrasound image; (**A2**) diagrammatic representation of (**A3**) post-test ultrasound image. Subcutaneous pitting (**B1**–**B3**). Both tissue layers (**C1**–**C3**).

The duration of the pressure phase of the test was 60 s, and this was most often the timepoint at which the maximum pitting depth was identified. If the pressure phase of the test was to continue longer than 60 s, the tissue may have continued to pit further. Lymphoedema tissue was slower to reach the maximum pitting depth than contralateral sites for dermal (not significant) and subcutaneous tissue layers (*p* = 0.032). Tissue layer involvement in pitting varied with either or both the dermis and subcutis involved (Figure 2). Pitting lymphoedema sites involved dermal and subcutaneous tissue layers more often than pitting unaffected sites (*p* < 0.010) (Table 1).

LOCAL classification groups are defined by objective measurements to indicate lymphoedema tissue compositional change. No oedema refers to sites that do not exhibit any lymphoedema characteristics (*n* = 6/45). The Early Fluid category refers to tissue that exhibits extracellular fluid increase without compositional changes (*n* = 13/45). Whereas the High Fluid+ category refers to tissue that exhibits fibro-fatty changes in addition to high extracellular fluid volume (*n* = 14/45). Lastly, the Low Fluid+ group refers to tissue that exhibits fibro-fatty compositional changes with low extracellular fluid volume (*n* = 12/45) [12].

**Figure 3:**
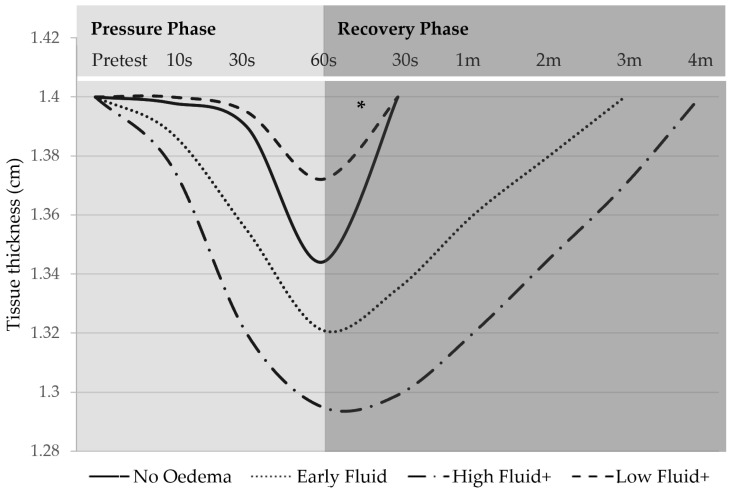
Timeline of tissue thickness change throughout pressure and recovery phases of the pitting test: Representation of LOCAL category trends. Graph generated using LOCAL subgroup data including: Average tissue thickness change at 60 s; average recovery time; the trend observed over the duration of pressure and recovery phases for sites approximating 1.4 cm cross-sectional thickness prior to pitting test. Pitting depths are representative of the data collected with the weight of an ultrasound standoff in situ reducing the magnitude of the measured pit. s—seconds; m—minutes. * The first measurement was taken 30 s into recovery after the pitting test; No Oedema and Low Fluid groups could have recovered beforehand.

**Figure 4:**
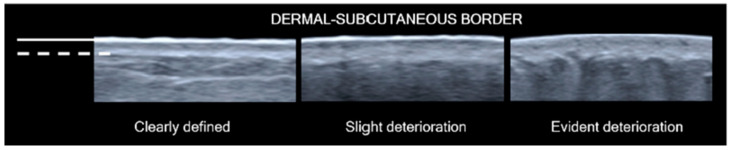
Greyscale ultrasound images of dermal-subcutaneous tissue layer border integrity. Borderlines of the epidermis (solid line) and dermal-subcutaneous border (dotted line) are indicated in white markings. A clearly defined border appears as a bright, uninterrupted hyperechoic line that separates the tissue layers with minor undulation along the border length (**left**). Slight border clarity deterioration is demarcated by a loss in contrast to echogenicity between tissue layers (**middle**). Evident deterioration of border integrity with the borderline interrupted by large serrations extending between tissue layers (**right**).

Changes in tissue thickness were observed across LOCAL categories (Figure 3). Sites in the “No oedema” category registered nil to low levels of pitting. For the “No oedema” sites with a pitting effect, the tissues had the same tissue layer involvement and similar pitting depth to the matched contralateral sites and recovered within 30 s. Lymphoedematous sites in the “Early fluid” category and the “High fluid+” groups demonstrated greater tissue layer involvement in pitting, greater depth of pitting, and an extended recovery period compared to other LOCAL categories. In contrast, 75% of the “Low fluid+” group demonstrated reduced or no pitting at the lymphoedema site, less tissue layer involvement than contralateral comparison sites, and a short tissue recovery period within 30 s post-test. The deepest pitting occurred in tissues where there was deterioration in dermal-subcutaneous border clarity (Figure 4). Border deterioration was more frequent in lymphoedema sites (*p* < 0.001), although not present in all pitting tissue, and there was no clear relationship with LOCAL classification (Table 2).

## 4. Discussion

This study investigated how lymphoedema tissue responds to sustained pressure over 60 s throughout the pitting test in order to guide standardisation of the test. The results corroborate previous research indicating lymphoedema tissue responds more slowly to applied pressure [7,8] and therefore requires a longer test duration to obtain pitting indentation than other conditions that exhibit pitting, such as cardiac failure. We found that low-grade pitting commonly occurs in normal tissue, and the characteristics of pathological pitting are best identified using a known unaffected comparative site. To the author’s knowledge, this is the first study to verify specific components of the dermal and subcutaneous tissue layers that contribute to pitting induration speed and depth.

The staged pitting test technique used in this study allowed evaluation of pitting initiation and change over time. Sustaining pressure for 10 s was not long enough for some lymphoedema tissue to initiate a pitting effect; 30 s of pressure demonstrated a greater likelihood of a pitting effect in tissues; and at 60 s, we were confident that all sites that could exhibit pitting had enough time to do so. The 60 s test also allowed time for differences between lymphoedema-affected and unaffected tissue to become apparent. Tissues that demonstrated pitting within 10 s often increased pitting depth over the remainder of the test and took longer to recover. Regardless of pitting depth, tissue less amenable to pitting recovered quicker than readily pitting tissue. These findings support a pitting test duration longer than 10 s to enable tissues that can exhibit a pitting effect to do so, ensuring potential pitting is detected. A test longer than 60 s was expected to increase tissue recovery duration and increase the likelihood of non-oedematous tissue developing an indent. Neither of these outcomes yields further information, even if pitting depth continued to increase over the longer test period. These factors would require further investigation in a future study.

The pitting attributes of lymphoedema tissue are recognised to change with clinical progression as a differentiating criterion between lymphoedema stages [2]. Lymphoedema pitting is different from pitting in other conditions, where pitting initiation [7] and response to pressure [8] are prolonged. The pitting test technique is critical for lymphoedema assessment as the tissue does not typically respond immediately to localised pressure, as may be observed in some venous impairments. Consequently, an effective pitting assessment for cardiac failure-induced venous oedema of moderate pressure applied for 5 s, is unlikely to translate to an effective test in lymphoedema tissue that can take greater than 4 s to initiate change [7]. 

Prior to this research, tissue pitting was understood to be associated with subcutaneous oedema [5]. However, our ultrasound investigations show that lymphoedema can affect the dermal and subcutaneous tissue layers, and either or both tissue layers can be involved in pitting. In addition, greater pitting depths involved more tissue layers but did not correlate with oedema-specific measurements. We found that tissue layer involvement, pitting depth, and ease of pitting were not solely attributable to oedema volume.

As localised pressure was applied to the tissue layers, the force facilitated extracellular fluid (ECF) movement to the immediate surrounding tissue, as observed in post-pitting ultrasound images. The amount of ECF present in the tissue layers contributed to the movement of fluid. For example, if the tissue layers comprised a large volume of free-fluid oedema, the tissue would yield to pressure and form a pitting indent within the first 10 s of the pitting test. However, pitting was not attributable to ECF alone, as evidenced by BIS results, and we discovered that the extracellular matrix (ECM) structure of the tissue layers also influences the pitting capability of tissue, as demonstrated by LOCAL categorisation and d-s border integrity. Moreover, it is highly probable that external factors known to influence skin health, such as age, smoking, and sun exposure [13], in addition to diet and medications [14], could also contribute to pitting. 

In this study, the subjective appearance of the d-s border in ultrasound images was a tissue characteristic that influenced pitting depth, although deterioration of the structure was not unique to lymphoedema sites. We perceived the border integrity as an indicator of the potential structural strength of the tissue layer, where deterioration of the border enabled the dispersion of pressure between tissue layers during the pitting test pressure phase. When the d-s border was visually intact in pitting tissue, ECF was noted to shift towards the tissue adjacent to the test zone while still being retained within the border confines. This effect was best illustrated in tissues with dermal-only or subcutaneous-only pitting. Deterioration in the d-s border was marked by a visible loss in the definition of the structural borderline, with evident undulations and serrations observed on ultrasound imaging. Cellulite deposition in non-lymphoedema tissue [14] and advanced stage lymphoedema tissue [15] is known to express this tissue characteristic. Sites that exhibited both d-s deterioration and increased ECF volume registered the greatest pitting depths in the study. Furthermore, ultrasound elastography imaging of the same dataset, presented in another article by the same authors, emphasises the influence of d-s border integrity on the distribution of tissue stiffness [16]. The significance of d-s border deterioration deserves further investigation as a possible indicator of lymphoedema progression and to determine clinical meaning. 

Finally, the compositional structure of the ECM was indicated as a factor in pitting, as observed in ultrasound imaging and LOCAL grading. Fibrotic and fatty deposition were observed to influence tissue compressibility during the pitting pressure phase, which is thought to be associated with the density of the restructured tissue and the space it occupies within the tissue layer. The movement of ECF was observed to be limited by the physical barrier produced by the pathological changes occupying the region of interest on ultrasound scanning, thereby limiting pitting depth capability. In sites with late-stage disease where pathological changes encompassed the full cross-section of the tissue layer, the abnormal tissue acted to reduce space for movement of the ECF in addition to resisting compression. 

As shown in this study, results from the novel LOCAL categorisation of lymphoedema tissue support the relationship between the known pathophysiology of lymphoedema and pitting expectations [2]. Initially, when the predominant change is an accumulation of free fluid, pitting is evident and increases with greater volume. With lymphoedema progression, the density and proportion of fibrotic and fatty deposition increase, the ECF volume appears to reduce, and the lymphoedema tissue resists localised pressure, becoming non-pitting oedema [2]. A factor not currently accounted for is the role of the d-s border in tissue layer structure and its effect on the dispersion of pressure between tissue layers, regardless of indicators of oedema and tissue layer thickness.

### 4.1. Recommendation

There is known variability in pitting test techniques between health professionals in the current unstandardised form [3]. The results herein demonstrate that a staged method enables the identification of pitting initiation, pitting depth, and recovery time. Applying the pitting test pressure for 60 s allows tissue that is able to exhibit pitting to do so and allows time for differences between lymphoedema-affected and unaffected tissue to become apparent. The way that tissues pit over 60 s was also shown to be suggestive of tissue layer characteristics pertinent to lymphoedema evaluation.

The staged pitting test technique modelled in this study is recommended for lymphoedema assessment until future research can further guide clinical practice. The authors recommend using firm thumb pressure for a total of 60 s pressure duration in a staged method and observing recovery for a minimum of 60 s. The staged pitting test is applied clinically by maintaining a pressure of approximately 5 kg using a broad thumbprint area of 5 cm^2^ over the region of interest. The pressure is applied for 10 s and the thumb is lifted to observe the effect; pressure is reapplied at the same site for a further 20 s and the thumb is lifted again to observe the effect (30 s total pressure); re-apply pressure for a further 30 s and observe the effect (60 s total pressure); and finally, observe tissue recovery for a minimum of 60 s. The method described uses set timepoints for repeatability; it may be modified to a timeframe where the tissue is felt to respond and an observed effect is noted; however, the same timeframe must be used on the contralateral unaffected site for a comparative reference. Tissue density and client comfort should guide the clinical application of this recommended pitting test method with respect to the firmness of the thumb pressure applied.

Implementing a staged pitting test technique into clinical practice will improve the validity and accuracy of identifying “pitting” in lymphoedema tissue. This simple test is able to be performed by any health professional to complement the initial assessment of a presentation for diagnosis and staging, evaluate treatment effectiveness, and identify clinical progression in lymphoedema when performed consistently.

### 4.2. Limitations

The weight of the standoff used for ultrasound imaging diminished the pitting depth magnitude, creating what appeared on visual observation to be a stretch of the pitting test zone and compression of the tissue on either side of the pit. Further research on standardising the pitting test in various pathologies is required, with this study only reporting on within-subject normal and lymphoedema tissues. In addition, further investigations of the movement of ECF would assist in understanding the tissue response to the pitting test. Limitations to the research method include the relatively small sample size that did not include lymphoedema presentations with extreme severity. There was a potential for blinding bias, with the research team unable to be blinded from lymphoedema affected tissue and unaffected tissue.

## 5. Conclusions

The pitting attributes of tissue as measured in this study appeared to be influenced by extracellular fluid and the structural extracellular matrix of the dermal and subcutaneous tissue layers, including border integrity. Lymphoedema pathophysiological abnormalities of fibrosis and adiposis contribute to differences in the manner in which tissue responds to and recovers from the pitting test. The authors make recommendations on how the pitting test can be standardised.

## Figures and Tables

**Table 1:** Tissue layer contribution to pitting effect for lymphoedema and contralateral sites.

Tissue Layer	Lymphoedema (*n* = 45)	Contralateral (*n* = 45)
Neither	3	14
Dermal only	7	9
Subcutis only	11	15
Both layers	24	7

**Table 2:** Tissue layer border integrity grading for lymphoedema-affected and unaffected sites.

Border Integrity Grading	Lymphoedema (*n* = 45)	Contralateral (*n* = 45)
Clearly defined	11	28
Slight border clarity deterioration	29	17
Evident border clarity deterioration	5	0

## Data Availability

The data presented in this study are available on request from the corresponding author, J.S.
